# Field investigation of the bearing behavior of grouted gravel pile composite foundations in Karst Terrain

**DOI:** 10.1371/journal.pone.0348701

**Published:** 2026-05-13

**Authors:** Lingzhi Zhang, Changhe Yan, Chunyong Jiang

**Affiliations:** 1 College of Materials and Environmental Engineering, Chongqing Polytechnic University of Electronic Technology, Chongqing, China; 2 China Railway Engineering Design Consulting Group Co., LTD, Beijing, China; 3 College of Civil Engineering, Chongqing University, Chongqing, China; Henan Polytechnic University, CHINA

## Abstract

The extensive underlying karst in southwestern and southern China presents significant challenges for deformation control in railway and highway subgrades. This paper proposes an integrated technology combining grouted gravel piles with the treatment of underlying karst dissolution fractured zones, along with the requisite construction machinery and procedural sequence. The technology is implemented and validated through a railway subgrade project, involving a series of field investigations. The grouting-induced improvement mechanism in the surrounding soil is revealed through the evolution of lateral earth pressure and pore water pressure. The bearing behavior and load-transfer mechanism of a single pile are characterized by load-settlement curves, the distributions of axial force and shaft friction, and the load-sharing ratio. Finally, the effectiveness of this technology for the composite foundation is confirmed by field performance data under embankment loading, including settlements at the pile head and various soil depths, stress distributions on pile heads and adjacent soils, pore water pressures at different depths, and lateral displacements at the embankment toe.

## 1. Introduction

Soft soils, primarily composed of mucky and silty clay, are extensively distributed throughout southwestern and southern China. These soils exhibit high compressibility and low bearing capacity, predisposing them to pronounced time-dependent settlement under sustained loading conditions [1–4]. Furthermore, intensive karstification in these regions has resulted in the widespread development of karst dissolution fractured zones and even cavities beneath the overlying soft soil [5–6]. These fractured zones contain abundant fissure networks, and groundwater flow through them can exacerbate rock fragmentation [7–8]. The interplay between the low-bearing-capacity soft soils and adverse conditions within these fractured zones, such as fissure networks and groundwater flow, significantly increases the risk of differential settlement and even collapse. These complex geotechnical conditions pose formidable technical challenges for deformation control in railway and highway infrastructure projects [9].

Multiple improvement methodologies have been established for soft soil, which can be categorized by reinforcement mechanism into several types, including pile-supported composite foundations [10–13], drainage consolidation techniques [14], dynamic compaction and replacement methods [15–16], and chemical stabilization [17]. The pile-supported composite foundation technique enables efficient load transfer through synergistic interaction between pile elements and surrounding soil, forming a composite foundation that exploits the high load-bearing capacity of piles while mobilizing the inherent strength of native soil. This methodology has demonstrated particular effectiveness in settlement control and engineering stability assurance. Recent advancements have led to the development of numerous variants, including CFG (cement-fly ash-gravel) piles [18], jet grouting piles [19], precast tubular piles [20], cast-in-place piles [21], XCC (X-section cast-in-place concrete) piles [22], and grouted gravel piles [23]. For bridge foundation engineering in karst terrain, it was typically necessary to assess bedrock stability, which could entail traversing cavities. However, when dissolution fractured zones were encountered, this approach incurred prohibitive construction costs [24–25].

The grouted gravel pile technique was originally developed by Liu et al. [26]. It involves injecting grout through pre-installed pipes to bind aggregate and the surrounding soil, forming grouted gravel piles [23]. The simultaneous solidification of the grout and interlocking of the aggregate create a synergistic mechanism that enhances both the pile stiffness and the properties of the adjacent soils [27]. Gu et al. [28] proposed that settlement control in these composite foundations resulted from the integrated response of the pile elements, the grout-improved zones, and the surrounding soil mass. Through field investigations incorporating cone penetration and vane shear tests, Zuo et al. [29] substantiated the soil improvement effect and delineated the spatial distribution and extent of grout influence by comparing pre- and post-construction soil properties. Zhang et al. [30–31] employed transparent soil modeling combined with particle image velocimetry (PIV) analysis to quantify soil displacement induced by grout injection, elucidating soil compaction as the underlying improvement mechanism in the surrounding soil. Wen et al. [32] conducted field investigations to evaluate the performance of grouted gravel pile composite foundations, analyzing vertical and lateral deformations, pile-soil stress ratios, and pore pressure responses, which demonstrated the efficacy of the technology in controlling post-construction settlement. Chen et al. [33] performed comparative field tests examining settlement monitoring data and ultimate bearing capacities under different improvement methods including grouted gravel piles, vacuum preloading, and plain concrete piles, confirming the superior settlement control performance of grouted gravel piles.

When construction activities encountered fractured zones, pre-grouting treatment was conventionally employed prior to the commencement of main structural works [34–35]. However, this conventional two-stage approach reveals significant limitations when confronting the distinctive geological profile characterized by overlying soft soils underlain by karst dissolution fractured zones. The segregated treatment of fractured zones and soft soil strata as independent engineering phases results in protracted construction timelines and operational inefficiencies.

Recognizing the potential for integration between grouting in karst dissolution fractured zones and the column formation process of grouted gravel piles, this study develops an integrated grouting technology that enables the simultaneous stabilization of soft soil and the underlying karst dissolution fractured zones. The construction machinery and procedural sequence for this technology are introduced. To verify the effectiveness of this technology, a railway subgrade project is used for implementation, and a series of field investigations are conducted to analyze the grouting-induced improvement mechanism in the surrounding soil, the bearing behavior and load-transfer mechanism of a single pile, and the performance of the composite foundation under embankment loading.

## 2. Integrated grouting technology for grouted gravel piles and karst dissolution fractured zones

**Fig 1:**
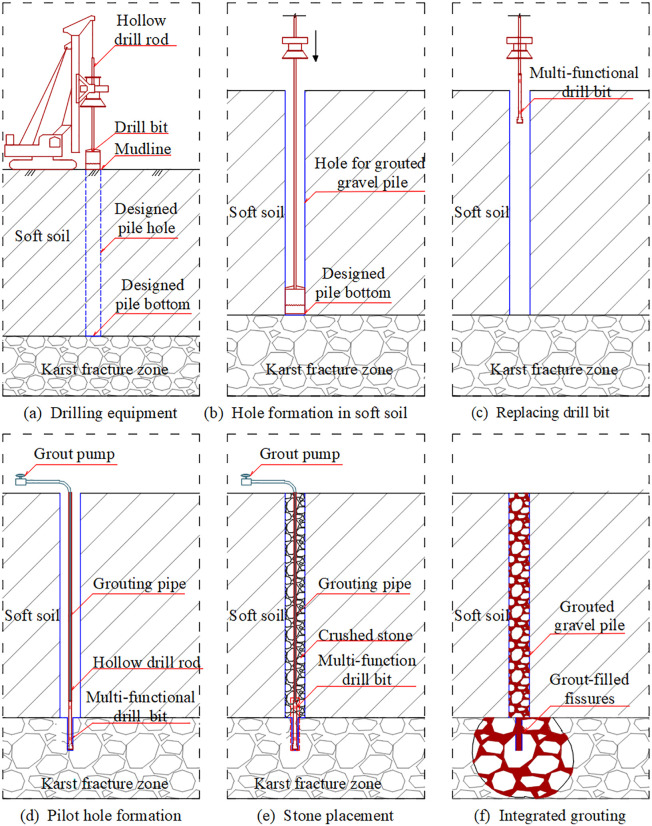
Integrated construction equipment and critical processes.

The construction equipment and critical processes for the integrated grouting technology, which combines grouted gravel piles with the treatment of underlying karst dissolution fractured zones, are illustrated in Fig 1. The construction procedure was carried out as follows:

(a) The drilling rig was positioned at the designated borehole center according to the engineering drawings.
(b) A specialized auger was used to advance the gravel pile borehole through the soft soil, forming a hole with a diameter of 0.4–0.8 m. Drilling was terminated upon reaching the upper boundary of the karst dissolution fractured zone.
(c) The drill bit was then replaced with a multi-functional tool capable of both drilling and grouting. This tool was used to advance a grouting pilot hole through the karst dissolution fractured zone until the predetermined target depth was reached.
(d) The grouting pipe assembly was installed, with the grouting pump and delivery lines connected through the hollow drill rods. The target grouting pressure was preset on the grouting recorder prior to injection.
(e) The pile borehole was filled with specifically graded crushed stone to form the skeletal structure of the grouted gravel pile.
(f) Regulated by the grouting recorder, the grouting pump injected grout into the karst fissures. Upon completion, the drill rods were raised to continue grouting through the gravel pile cavity until the entire integrated grouting process was completed.
(g) Supplementary crushed stone was then introduced, followed by compensatory grouting until the pile borehole was fully filled.

Table 1 summarizes the main differences between the proposed integrated grouting technology and the conventional method of pre-grouting followed by pile installation. The conventional method requires separate drilling for grouting holes in the karst dissolution fractured zone and for pile holes in the soft soil, along with a two-stage grouting process. In contrast, the integrated grouting technology enables continuous drilling through both the soft soil and the karst dissolution fractured zones, as well as continuous grouting that simultaneously treats the gravel pile and the fractured zone. Unlike the conventional method, which requires repeated repositioning, the proposed technology treats both zones with only a single repositioning of the equipment. These features streamline the construction sequence, reduce construction time, and improve operational efficiency. Furthermore, the reinforced fractured zone and the grouted gravel pile form an integrated whole, which offers significant potential for enhancing the overall mechanical performance of the composite foundation.

**Table 1:** Comparison between the integrated grouting technology and the conventional method.

	Conventional method	Integrated grouting technology
Phase	Phase Ⅰ: Pre-grouting Phase Ⅱ: Pile installation	Single continuous phase
Equipment	1 drilling rig and 1 drill bit per phase	1 drilling rig and 2 drill bits
Equipment positioning	Single positioning per phase	Single positioning for the whole process
Drilling	Phase Ⅰ: Grouting hole (in karst fractured zones) Phase Ⅱ: Pile borehole (in soft soil)	Integrated drilling for both pile and grouting holes
Grouting	Phase Ⅰ: Karst fissures Phase Ⅱ: Gravel pile cavity	Integrated grouting for both karst fissures and gravel pile cavity

## 3. Site characterization and testing protocol

### 3.1. Geological conditions

This study was a field trial conducted in direct conjunction with a railway engineering project in China. All field investigations, drilling, grouting operations, and installation of monitoring instruments were carried out during the construction phase of the project, within the specified engineering section (DK123 + 025 to DK123 + 400). The composite foundation for a railway station is situated within the Guangxi Zhuang Autonomous Region, China. The subgrade section DK123 + 025.0 to +400.0 was reinforced with grouted gravel piles. Core drilling investigations revealed extensive karst dissolution fractured zones beneath the overlying soft soil.

**Fig 2:**
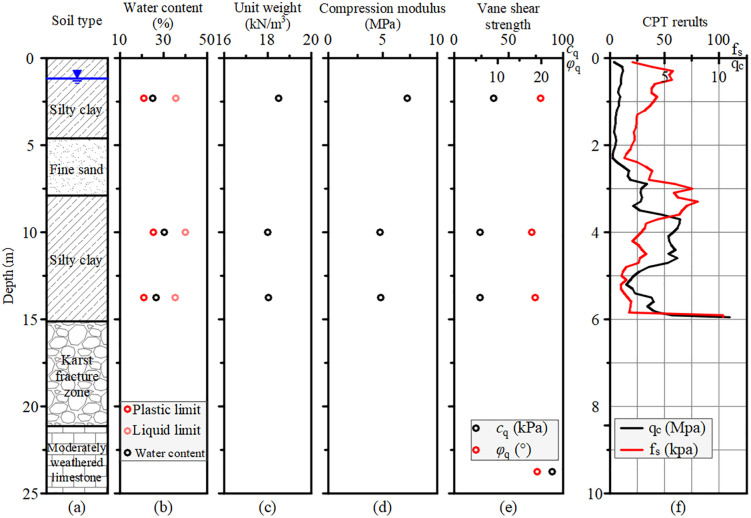
Soil profile and geotechnical properties at test section DK123 + 315.

Fig 2(a) illustrates the stratigraphic sequence at test section DK123 + 315 from the ground surface downward. The soil profile consisted of 4.60 m of stiff-plastic silty clay layer, underlain by a 3.30-m-thick fine sand layer and a 7.22-m-thick soft-plastic silty clay layer. Below these overlying soils, a 6.00-m-thick karst fractured zone composed of fragmented limestone exhibits significant dissolution features, underlain by moderately weathered limestone.

Laboratory tests were conducted to determine the geotechnical properties, with results summarized in Figs 2(b) to 2(e). The silty clay layers exhibited low to medium plasticity with natural water content ranging from 25.1% to 30.4%. The compression modulus values were 7.25 MPa for the stiff-plastic silty clay and 4.78 MPa for the soft-plastic silty clay. Quick direct shear tests yielded cohesion and internal friction angle values of 36.20 kPa and 19.86° for the stiff-plastic silty clay, 24.50 kPa and 18.10° for the soft-plastic silty clay layer, and 90 kPa and 19.00° for the moderately weathered limestone. Additionally, a cone penetration test (CPT) was conducted prior to construction, with results provided in Fig 2(f).

### 3.2. Layout of testing and monitoring instrumentation

#### 3.2.1. Design scheme of composite foundation.

**Fig 3:**
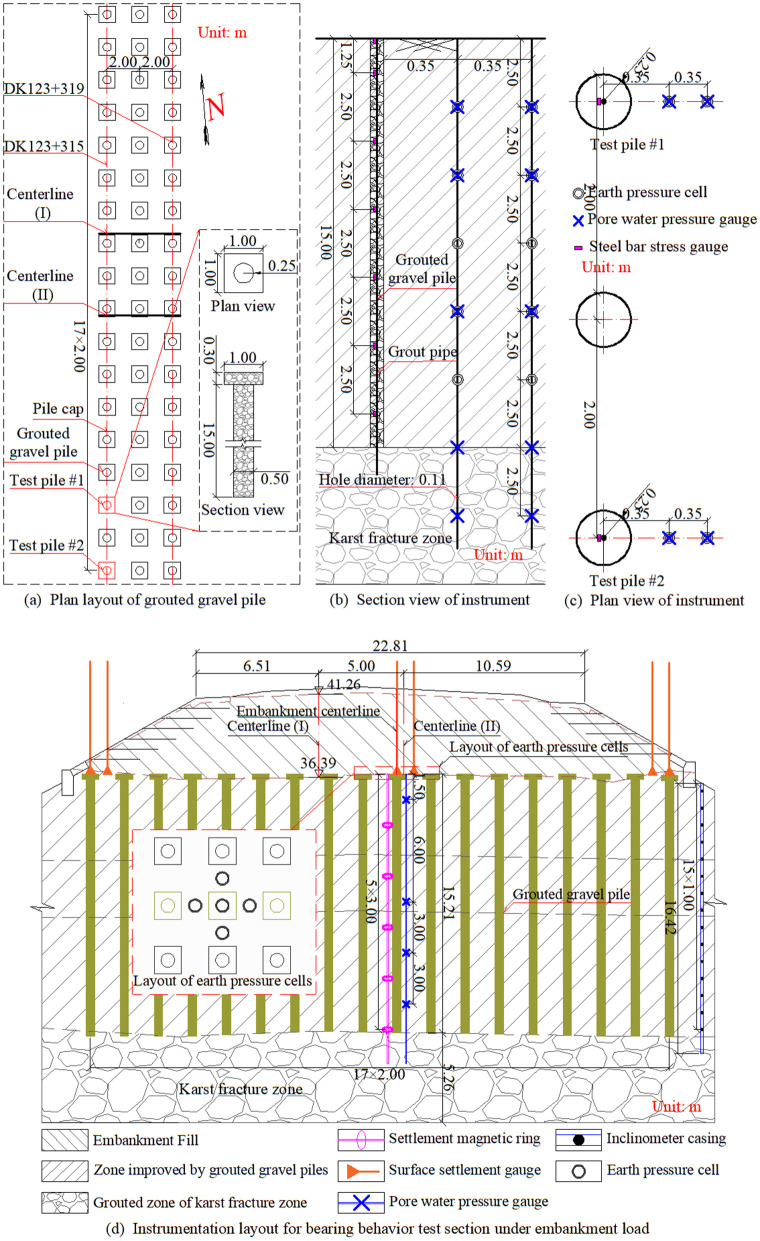
Design scheme and instrumentation layout.

The grouted gravel piles were constructed using crushed stone aggregate with particle sizes of 16–32.5 mm. The piles, with a diameter of 0.5 m and an embedment depth of approximately 15.0 m, were arranged in a square pattern at 2.0 m center-to-center spacing, as detailed in Fig 3(a).

Grouting was performed through 25-mm-diameter pipes using a cement-sand-water mix ratio of 1:1.2:0.6 under a constant pressure of 2.4 MPa. The grouting process was carried out continuously, with the grouting pipe being lifted progressively during injection. The average total grout consumption was approximately 4800 kg, forming the integrated composite of the reinforced fractured zone and the grouted gravel pile. In sections underlain by karst dissolution fractured zones, the integrated grouting technology achieved a consolidated improvement depth of 17.3–20 m.

Each pile was capped with a 0.3-m-thick C35 reinforced concrete slab measuring 1.0 m × 1.0 m. The areas between the piles were backfilled with compacted crushed stone. A 0.6-m-thick gravel cushion, reinforced with two layers of biaxial polyester geogrid, was then placed over the pile caps. The embankment was then constructed using layered filling methods on the reinforced foundation, as shown in Fig 3(d).

#### 3.2.2. Testing program.

A systematic field investigation was conducted to evaluate the performance of the integrated grouting technology in foundation reinforcement. Three experimental components were implemented:

(1) Grouted Gravel Pile Formation

As shown in Fig 3(a), test piles #1 and #2 were employed to investigate the formation characteristics of grouted gravel piles. Key monitoring parameters included the evolution of lateral earth pressure and pore water pressure during grouting operations.

(2) Single Pile Static Load Test

Test piles #1 and #2 were used to investigate the load-bearing behavior of single piles under static loading. Monitoring focused on pile head settlements and the distribution of axial forces along the piles.

(3) Composite Foundation Performance under Embankment Loading

As shown in Fig 3(d), the overall performance of the composite foundation at section DK123 + 319 was monitored under embankment loading conditions. Comprehensive monitoring included soil settlements at different depths, pile head settlements, stress distributions at pile heads and adjacent soils, pore water pressure at various depths, and lateral displacements at the embankment toes.

#### 3.2.3. Instrumentation Arrangement.

(1) Grouted Gravel Pile Formation and Single Pile Static Load Test

Figs 3(b) and 3(c) detail the instrumentation layout for monitoring grouted gravel pile formation and the single pile static load test. The instrumentation arrangement was identical for both test piles. Sensor clusters for monitoring grouted gravel pile formation were installed at radial distances of 600 mm and 950 mm from the pile centerlines. Each cluster comprised five pore water pressure transducers and five earth pressure cells. Additionally, two sets of reinforcement stress gauges (14 units total) for the single pile static load tests were installed along a 16-mm-diameter steel bar with a length of 15 m. The gauges were spaced at 3 m intervals and connected using threaded couplers.

(2) Composite Foundation Performance under Embankment Loading

To investigate the load-response behavior of the composite foundation during embankment construction, the DK123 + 319 cross-section was instrumented as shown in Fig 3(d). Four pore water pressure transducers were installed at different depths along the embankment centerline. Six surface settlement sensors were deployed with three on pile heads and three on adjacent inter-pile soil. The pile head sensors were placed at one central and two edge locations. Additionally, five earth pressure cells were installed: one on the central pile head and four in the surrounding inter-pile soil arranged in four directions. A 16-meter-deep borehole, equipped with five magnetic rings at 3-m intervals, was installed near the centerline for layered settlement monitoring. An inclinometer casing was placed 1 m beyond the toe of the right embankment slope.

## 4. Test results

### 4.1. Analysis of grouting-induced column formation effects

#### 4.1.1. Lateral earth pressure.

**Fig 4:**
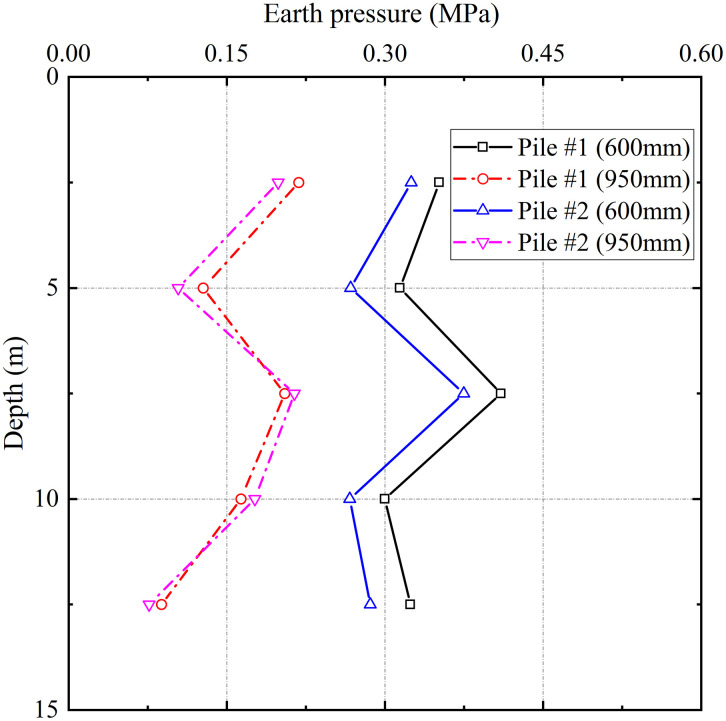
Lateral earth pressure distribution with depth and radial distance from the pile centerline.

Fig 4 illustrates the variation of lateral earth pressure with depth at radial distances of 600 mm and 950 mm from the centerlines of test piles #1 and #2 after grouting. The earth pressure distributions exhibit remarkable similarity between the two piles, indicating a consistent mechanism of grouting-induced soil compaction under identical geological conditions. Taking test pile #1 as a representative, the earth pressure in the near-pile zone (600 mm from the centerline) varied nonlinearly with depth, recording values of 0.35 MPa, 0.32 MPa, 0.51 MPa, 0.30 MPa, and 0.33 MPa at depths of 2.5 m, 5 m, 7.5 m, 10 m, and 12.5 m, respectively. In contrast, the earth pressures in the far-pile zone (950 mm away) were generally lower, measuring 0.22 MPa, 0.13 MPa, 0.41 MPa, 0.26 MPa, and 0.18 MPa at the corresponding depths. The maximum pressure in both zones occurred at 7.5 m depth, where the value at 600 mm (0.51 MPa) exceeded that at 950 mm (0.41 MPa) by 24.4%. These observations demonstrate the significant attenuation of radial soil compaction effects with increasing distance from the grout source.

#### 4.1.2. Pore pressure in the surrounding soil.

**Fig 5:**
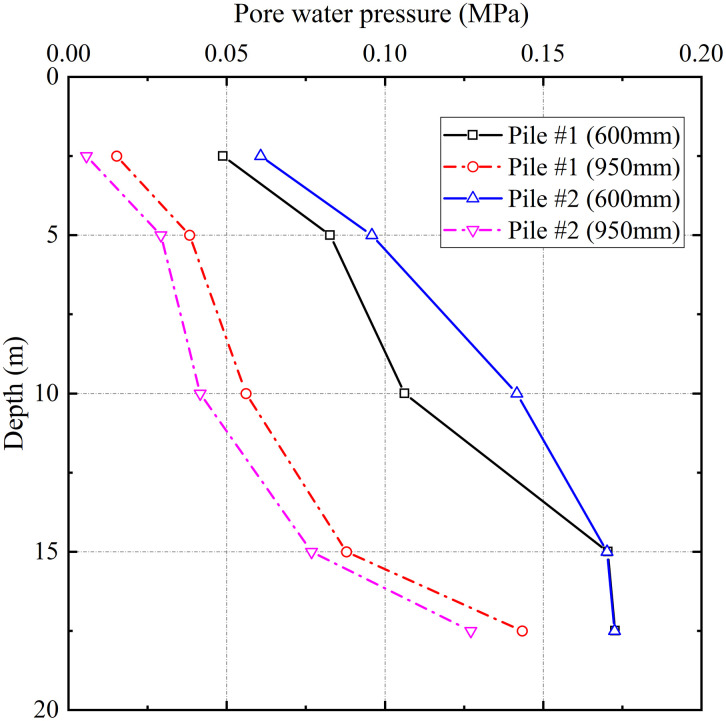
Pore water pressure distribution with depth and radial distance from the pile centerline.

Fig 5 presents the evolution of pore water pressures in the surrounding soil during grouting for test piles #1 and #2. The measurements revealed that pore pressure increased significantly with depth but decreased markedly with radial distance from the pile centerline. For test pile #1, pore pressure in the near-pile zone (600 mm from the centerline) increased from 48 kPa at 2.5 m depth to 173 kPa at 17.5 m depth, representing a 260% increase. In the far-pile zone (950 mm from the centerline), the corresponding values increased from 25 kPa to 163 kPa, corresponding to a 552% increase. The pore pressure in the near-pile zone consistently exceeded that in the far-pile zone at any depth. This spatial distribution pattern results from the combined effects of gravitational stress and grouting-induced permeation. In shallow depth, pore pressure dissipates more readily through both radial spreading and vertical drainage. In contrast, deeper depth exhibit reduced vertical permeability, resulting in dominant radial permeation and consequently greater pore pressure accumulation.

The distribution patterns of lateral earth pressure and pore water pressure indicate that the grouting pressure is transmitted through a dual-path mechanism: compression of the soil skeleton and elevation of pore water pressure.

### 4.2. Load-bearing behavior of single grouted gravel pile

#### 4.2.1. Load-settlement characteristics.

The single pile static load tests were conducted following the *Technical Code for Testing of Building Foundation Piles* (JGJ 106–2014), utilizing a counterweight platform reaction system. The slow maintained load method was employed throughout the testing process. Static loading was applied by hydraulic jacks positioned atop the test piles, while pile head settlements were measured with electronic dial indicators.

**Fig 6:**
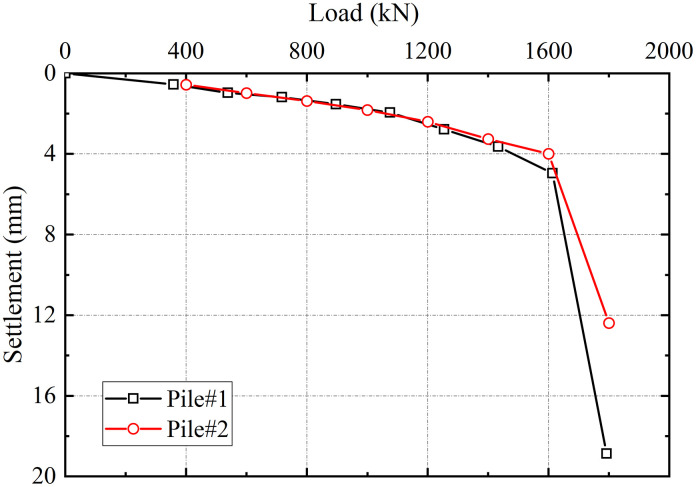
Load-settlement curves.

The load-settlement (*P*-*S*) curves for test piles #1 and #2 are presented in Fig 6. Both piles demonstrated nearly linear behavior under loads below 1000 kN, with settlements measuring approximately 1.70 mm at this load. As loading increased beyond this threshold, the *P*-*S* curves exhibited progressively nonlinear characteristics, with settlement increments accelerating under equivalent load increments. When the load exceeded approximately 1600 kN, both curves displayed a pronounced downward inflection, corresponding to settlements of 4.96 mm and 3.99 mm for test piles #1 and #2, respectively. The ultimate bearing capacity was determined as the load value at the characteristic point of pronounced downward inflection, yielding values of 1612.8 kN for test pile #1 and 1600 kN for test pile #2.

#### 4.2.2. Load transfer mechanism.

**Fig 7:**
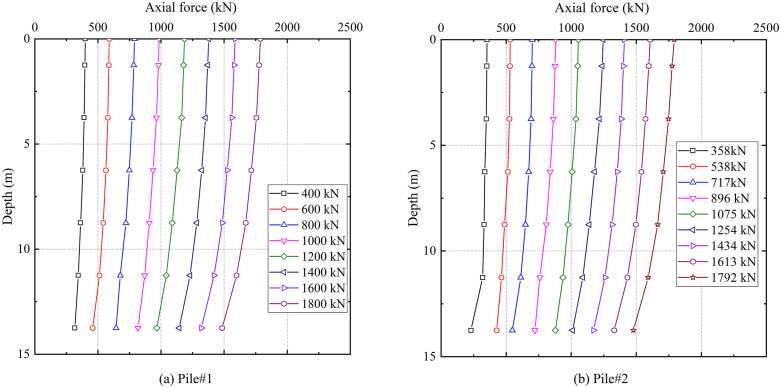
Axial force-depth curves.

The axial force distributions along the piles were determined from strain gauge readings obtained during static load testing. Figs 7(a) and 7(b) present the axial force-depth relationships for test piles #1 and #2. Both piles exhibited identical trends in axial force transmission, characterized by progressive attenuation from pile head to tip and significant increased at all depths under higher load levels. For test pile #1 at the depth of 13.75 m, under nine loading increments ranging from 358 kN to 1792 kN, the corresponding axial forces measured 228.92 kN, 427.02 kN, 548.01 kN, 721.56 kN, 878.76 kN, 1011.07 kN, 1171.79 kN, 1330.36 kN, and 1478.18 kN. The magnitude of axial force exhibited a direct correlation with the applied head load, demonstrating synchronous enhancement under increasing load.

**Fig 8:**
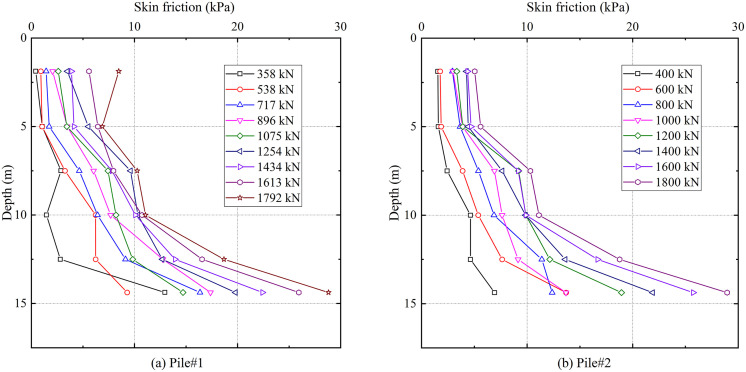
Skin friction distribution.

Figs 8(a) and 8(b) depict the shaft friction resistance distributions along test piles #1 and #2, respectively. Within the depth interval of 5–15 m, the mobilized shaft friction resistance demonstrated enhancement with increasing depth, reaching its peak value at the depth of 14.75 m. At any given depth, the shaft friction resistance increased under higher applied loads. This behavior reflects the coupling effect between initial geostatic stresses and pile-soil relative displacements.

**Fig 9:**
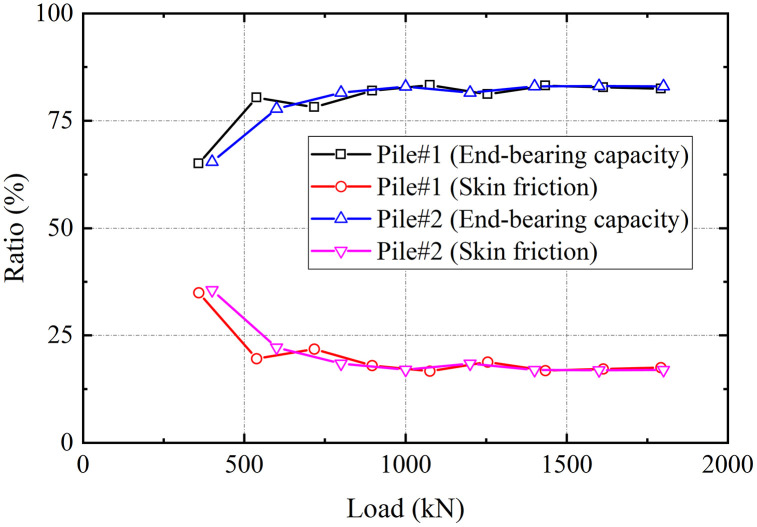
Load sharing ratio.

The applied static load at the pile head is equilibrated by the combined resistance of shaft friction and end-bearing. In this study, the end-bearing derives from the reinforced karst fractured zone. The proportional distribution of shaft friction resistance and end-bearing resistance to the total applied load, derived from measured axial forces and calculated shaft friction resistance data, is presented in Fig 9. Under the initial load, the end-bearing resistance and shaft friction resistance components accounted for 65% and 35% of the total load, for both test piles. As loading increased, the load transfer mechanism exhibits a notable redistribution. The end-bearing resistance contribution increases to 83% of the total load, with a corresponding decrease in shaft friction resistance to 17%, beyond which both values remain relatively constant. End-bearing resistance acted as the primary contributor to the overall bearing capacity during the entire loading process.

The load-settlement curve initially exhibits a distinct linear segment under small settlements, followed by a rapid increase in settlement that marks the onset of failure. End-bearing constituted over 80% of the total capacity, exhibiting locked-in synchronous development with shaft friction resistance that ensured efficient load transfer to the tip. The bearing behavior of grouted gravel piles in treated karst zones featured a distinct plunging failure, diverging from the gradual transition of fractured rock masses [29]. This behavior results from the grouted fractured zone gaining enhanced integrity and stiffness, instead of remaining loose and fragmented. This behavioral pattern, combined with the dominant end-bearing mechanism, substantiates the efficacy of the integrated grouting technology in substantially enhancing the load-bearing performance of karst dissolution fractured zones.

### 4.3. Composite foundation performance under embankment loading

#### 4.3.1. Surface settlement.

**Fig 10:**
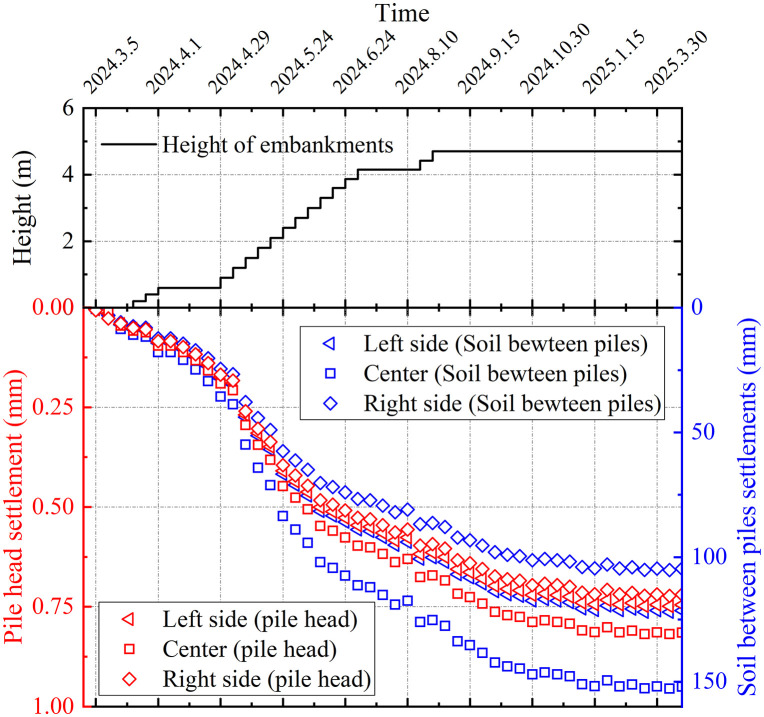
Surface settlements time-history curves for section DK123 + 319.

The settlement evolution during and after embankment placement serves as a critical indicator for construction sequencing and reflects the effectiveness of soft ground improvement. Fig 10 presents the time-history relationships between embankment fill height (*H*) and surface settlements at both pile heads and inter-pile soils for section DK123 + 319. The monitoring data revealed a positive correlation between settlement magnitude and height of the embankment, with greater settlements recorded at the centerline than at the edges. During initial construction stages, both pile heads and inter-pile soils exhibited rapid settlement trends, reflecting the instantaneous compression characteristics of shallow soft under additional stress. In subsequent phases, settlement rates demonstrated nonlinear attenuation with increasing differential settlement between piles and soils. The maximum settlements reached 0.82 mm at pile heads and 152.68 mm in inter-pile soils at the centerline, confirming the dominant load-transfer mechanism of the pile elements in the composite foundation.

#### 4.3.2. Stratified settlement.

**Fig 11:**
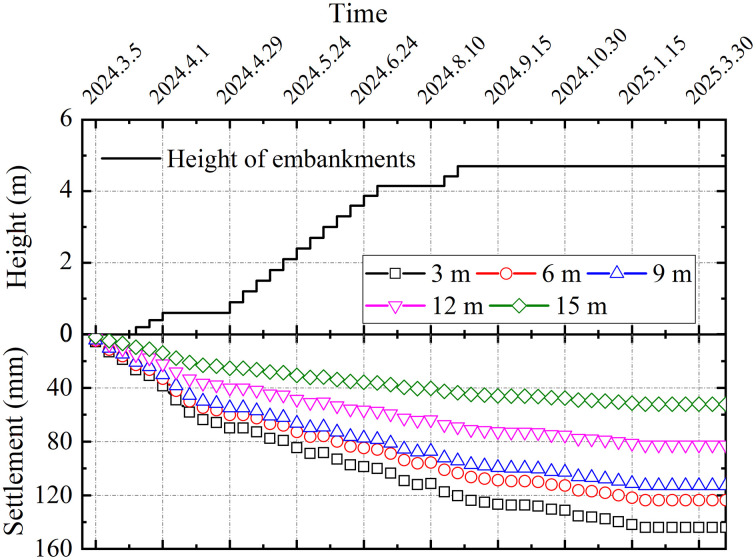
Stratified settlements time-history curves for section DK123 + 319.

Fig 11 presents the time-history relationship between embankment fill height and layered settlements at section DK123 + 319. A close similarity was observed between the settlement evolution of each soil layer and the surface settlement, with both paralleling the increase in fill height. Measured settlements exhibited reduction with depth, recording values of 143.99 mm, 123.64 mm, 112.88 mm, 82.48 mm, and 52.18 mm at depths of 3 m, 6 m, 9 m, 12 m, and 15 m. The settlement at 3 m depth (143.99 mm) approached the surface settlement (152.68 mm). Stratified settlement measurements indicate that compressive strains in the grouted gravel pile composite foundation under embankment load were largely confined to the upper-middle portions of the pile-soil interface.

#### 4.3.3. Lateral displacement.

**Fig 12:**
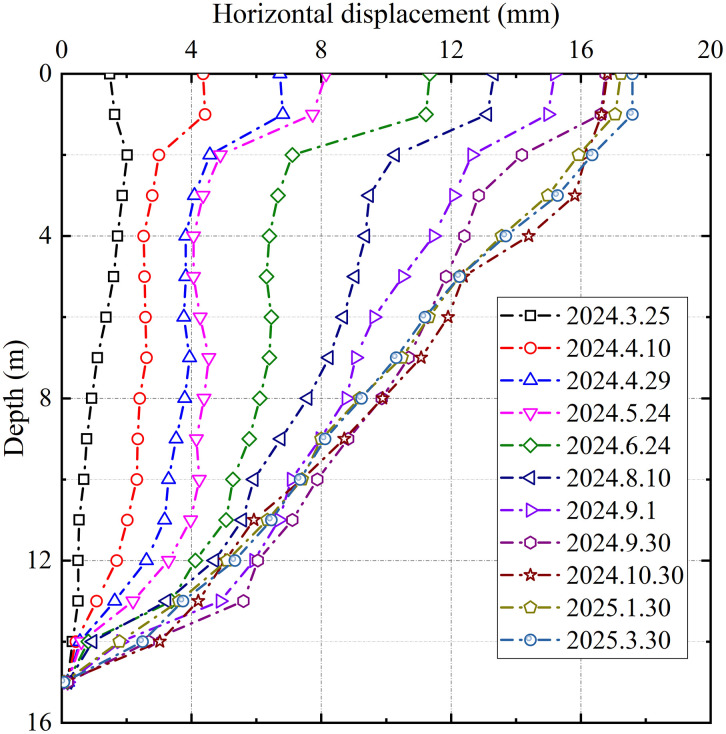
Horizontal displacements for section DK123 + 319.

Fig 12 presents the lateral displacements of the embankment foundation, as measured by an inclinometer installed at the slope toe. The reinforced zone, treated with grouted gravel piles, exhibited its maximum horizontal displacement (under 20 mm) at the ground surface, confirming that the deformation remained within safe limits. The displacement decreased smoothly with depth, and the absence of localized abrupt changes indicated no evidence of subgrade sliding failure. This behavior resulted from the lateral confinement provided by the pile rigidity, which distributed plastic deformations through group effect. The coordinated pile-soil deformation mechanism effectively suppressed potential shear band development, while the geogrid layer redistributed horizontal stresses through membrane action, mitigating stress concentration at the slope toe.

#### 4.3.4. Pore pressure.

**Fig 13:**
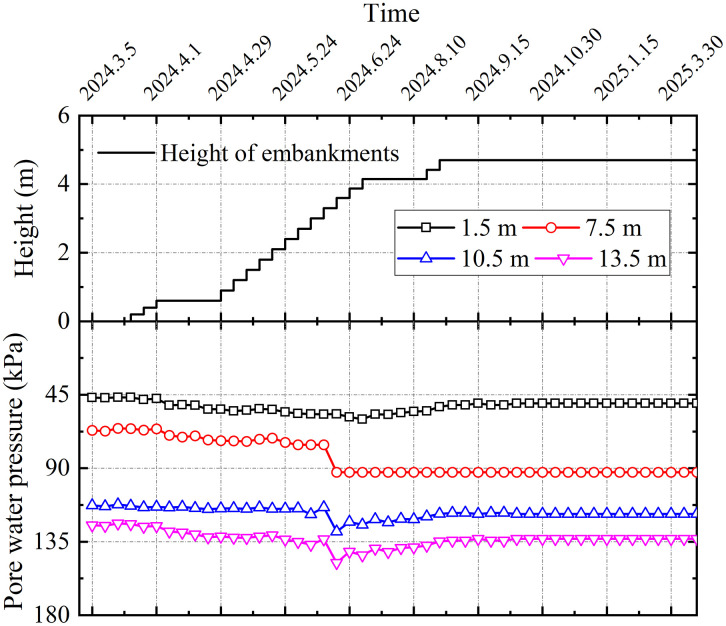
Variation of pore water pressure in section DK123 + 319.

To evaluate the foundation consolidation, pore water pressure transducers were installed at multiple depths to monitor pore pressure evolution and assess the degree of consolidation in each soil layer. Fig 13 presents the evolution of pore water pressure with embankment height at depths of 1.5 m, 7.5 m, 10.5 m, and 13.5 m in this investigation. During the initial stage, the recorded pore pressure increases remained relatively modest. As filling progressed, the pore pressure stabilized with negligible variation. In the final stage and after the completion of filling, a gradual dissipation of pore pressure was observed. This response is primarily attributed to the load transfer mechanism triggered by embankment construction, which involves cushion and soil-arching effects. Initially, the geogrid-reinforced cushion facilitated load distribution between piles and the surrounding soil. Upon reaching a critical embankment height, a pronounced soil-arching effect developed. The synergistic action of the reinforced cushion and soil arching effectively diverted the majority of the embankment load onto the piles, thereby limiting the increase in stress within the soft soil matrix. Consequently, in accordance with the principle of effective stress, the generation of excess pore water pressure was suppressed. Furthermore, the staged filling method allowed sufficient time for the excess pore water pressure induced by preceding layers to dissipate significantly.

#### 4.3.5. Soil pressure distribution.

**Fig 14:**
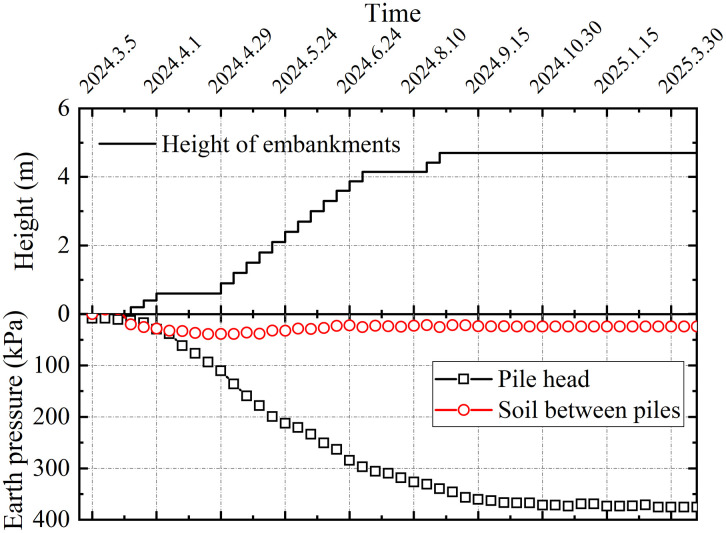
Variation of soil pressure in section DK123 + 319.

Earth pressure monitoring was implemented to characterize the load-transfer mechanism between piles and soil to analyze the behavior of the composite foundation and pile-soil interaction. Field measurements at section DK123 + 319, presented in Fig 14, demonstrate the evolution of earth stresses atop piles and adjacent soils with increasing embankment load. Monitoring data recorded progressive earth stress increased in both pile heads and the surrounding soil, with the pile heads exhibiting significantly greater increments in contrast to the adjacent soil. Upon completion of backfilling, the stresses on the pile heads and the inter-pile soil stabilized, indicating that the load distribution between these components had reached equilibrium and that the composite foundation was functioning in a coordinated manner. Owing to the cushion and soil-arching effects, the embankment load was transferred and concentrated on the pile heads. The measured stresses were approximately 375 kPa on the pile heads and 13 kPa on the inter-pile soil, indicating a significant stress concentration on the piles.

**Fig 15:**
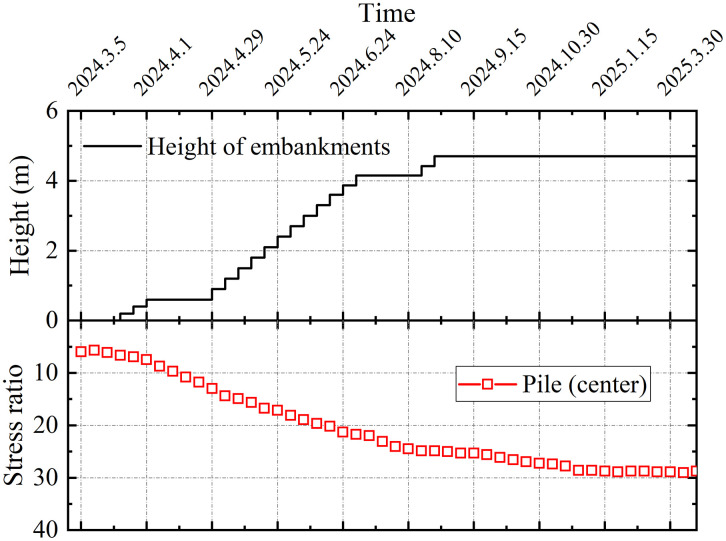
DK123 + 319 section pile-soil stress ratio.

Fig 15 shows the pile-soil stress ratio at the center of the subgrade (section DK123 + 319). A stress ratio of approximately 29 demonstrates the dominant role of the piles in load transfer, confirming the typical bearing behavior of a rigid pile-composite foundation.

**Fig 16:**
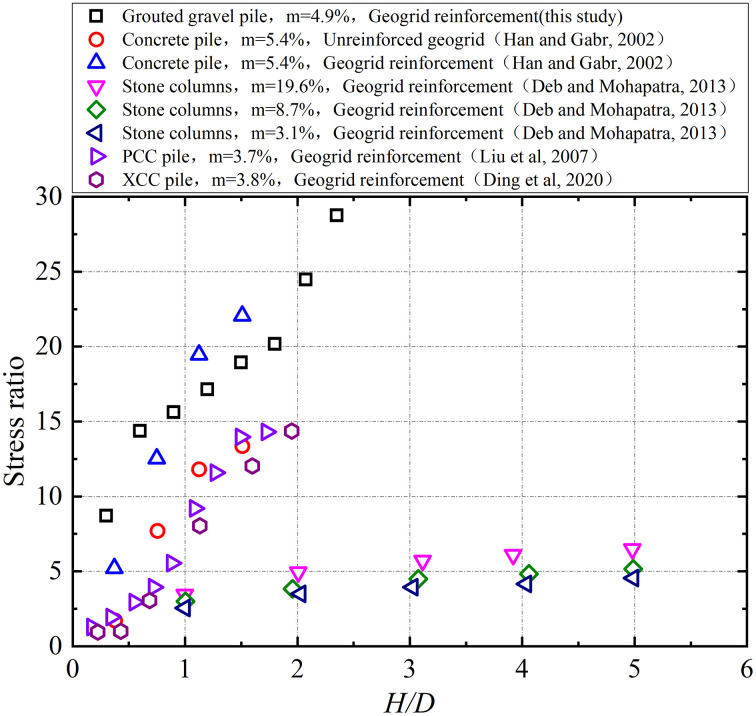
Comparison of stress concentration ratio with previous studies.

The field-measured pile-soil stress ratios for grouted gravel piles in this study are compared with established data from prior research in Fig 16, where *m* denotes the area replacement ratio. As shown in the figure, the results are grouped into two categories: those exhibiting rigid pile behavior and those exhibiting flexible pile behavior. The pile-soil stress ratio for rigid piles shows a consistent increase with the embankment height-to-width ratio (*H/D*), as demonstrated by concrete piles, PCC piles, XCC piles, and grouted gravel piles. The load-bearing mechanism of flexible piles (e.g., stone columns) differs, resulting in a relatively insensitive or limited increase in the pile-soil stress ratio with increasing *H/D*.

The non-uniform stress distribution between the pile and surrounding soil, induced by soil arching effects [36–39], can be quantified by the arching coefficient k expressed in Eq. 1.


$$k=\frac{\sigma_{\text{s}}}{\gamma H+p_{\text{0}}}$$ (1)

where γ represents the unit weight of the fill material, $p_{\text{0}}$ signifies the surcharge pressure applied on the embankment surface, and $\sigma_{\text{s}}$ indicates the earth pressure acting on the inter-pile soil.

**Fig 17:**
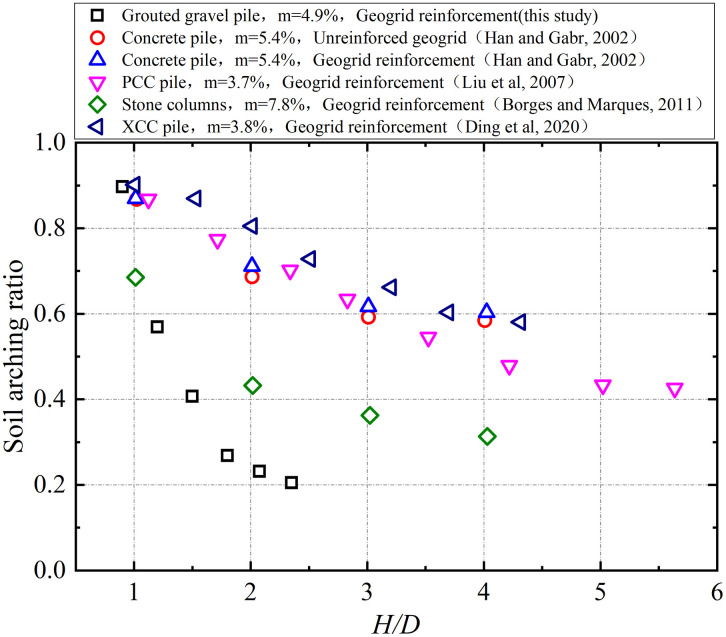
Comparison of soil arching ratio with previous studies.

The relationship between the soil arching coefficient and the *H/D* ratio for grouted gravel piles was validated against existing test results, as shown in Fig 17. The arching coefficient for the ground improved with grouted gravel piles demonstrated a nonlinear reduction with increasing *H/D*, decreasing to 0.21 upon completion of embankment placement. This value was lower than those of other pile types and closely matched results from the results reported by Borges and Marques [40] for jet grouting piles during the early filling construction. This correlation can be attributed to the jet grouted piles in Borges and Marques with great pile coverage, having a high area replacement ratio, small pile spacing, and relatively large pile stiffness, which collectively minimized the differential settlement between the piles and surrounding soil.

## 5. Discussion

While this study demonstrated the feasibility of the integrated grouting technology and elucidated the bearing mechanism of its composite foundation under specific conditions, several inherent limitations defined the applicability of the conclusions drawn herein.

(1) Karst dissolution fractured zones exhibit strong spatial variability [41], influenced by factors such as the thickness of the karst layer, the degree of rock fracturing, and the aperture and connectivity of fissures. Additionally, the geometry and strength of the grout-reinforced zones vary significantly with soft soil thickness, grout volume, and mix proportions. These variations affect both the bearing behavior of individual piles and the deformation characteristics of the composite foundation. The findings and conclusions presented in this paper are primarily applicable to geological conditions similar to those described in the case study. Future research can integrate multifunctional drilling tools with ultrasonic profiling capabilities to characterize the morphology of the fractured zones before and after grouting treatment. When applied to projects with notably different fracture characteristics, soil layer structures, or loading conditions, the technical approach should be accordingly adjusted and subjected to project-specific evaluation.
(2) The field monitoring in this study primarily covered the construction and a short-term post-construction phase of several months. This duration allowed for a thorough evaluation of the immediate effectiveness of the technology in controlling settlement during construction, ensuring stability, and assessing the completion of primary consolidation. However, the absence of long-term performance monitoring data over several years or even decades precludes a direct assessment of the long-term operational settlement of the composite foundation, the durability of the grouted bodies over time, and their performance evolution within the dynamic karst environment. In future work, long-term monitoring systems should be installed in critical projects. These systems would track the time-dependent variations in pile-soil stress, settlement, and groundwater level. Such monitoring would allow for a direct assessment of the long-term service performance and durability of the technology.
(3) This study examined the composite foundation response only under static loading during staged embankment filling. It did not consider the effects of long-term cyclic dynamic loads from train operation, instantaneous impact loads, or other accidental loads. Consequently, the conclusions regarding load transfer and deformation control are primarily applicable to embankment engineering conditions dominated by static loads. Future work will employ model tests or numerical simulations to investigate the dynamic response, cumulative deformation, and long-term stability of the composite foundation under complex loading conditions such as traffic-induced cyclic loads and seismic loads.

## 6. Conclusions

This study developed an integrated technology combining grouted gravel piles with the treatment of underlying karst dissolution fractured zones, followed by engineering implementation. The grouting-induced improvement mechanism in the surrounding soil, the bearing behavior and load-transfer mechanism of a single pile, and the performance of the composite foundation under embankment loading were elucidated through field investigation. The principal findings are summarized as follows:

(1) The lateral earth pressure and pore water pressure included by grouting decreased with increasing distance from the grout source, while pore water pressure increased with depth with depth. The grouting-induced improvement mechanism in the surrounding soil primarily involved soil skeleton compression and osmotic consolidation.
(2) The initial segment of the load-settlement curve exhibited pronounced linearity with minimal pile-head displacement. Upon reaching the ultimate bearing capacity, the curve transitioned into a characteristic plunge, yielding ultimate load capacities of approximately 1600 kN for single piles. The base resistance contributed over 80% of the total capacity, confirming efficient load transfer to the pile tip.
(3) During embankment construction, settlements at pile heads remained substantially smaller than those in the surrounding soils, while soil compression was predominantly concentrated within the upper-to-middle sections adjacent to the piles. Lateral displacements of soil strata remained below 20 mm, confirming the efficacy of lateral confinement provided by the composite foundation system.
(4) Substantially greater stresses developed on the pile heads compared to the surrounding soils, with the pile-soil stress ratio stabilizing at approximately 29, demonstrating typical rigid pile behavior. The differential settlements between grouted gravel piles and adjacent soils activated pronounced soil arching effects. The arching coefficient exhibited a nonlinear decrease with increasing *H/D* ratio, diminishing to 0.21 upon embankment completion, a value lower than those observed for other pile types.

## Supporting information

S1 Dataset Minimal data set for replicating the results of this study. (ZIP)
